# Age Differences in Preparedness for the Continuation of COVID-19: Important Roles of Social Support

**DOI:** 10.1093/geroni/igab046.1357

**Published:** 2021-12-17

**Authors:** Zhirui Chen, Zhen Cong, Daan Liang, Guofeng Cao

**Affiliations:** 1 University of Texas at Arlington, Arlington, Texas, United States; 2 University of Alabama, Tuscloosa, Alabama, United States; 3 University of Colorado Boulder, Boulder, Colorado, United States

## Abstract

This study examined the association between age and preparedness for the continuation of COVID-19. The moderation effects of three types of social support, namely, emotional, financial, and instrumental assistance were also tested. Using a sample of 443 adults in Dallas county which has the most confirmed cases in Texas, results of multiple linear regressions showed that compared to those aged between 18 and 64, older adults aged 65 and reported better preparedness for the continuation of COVID-19. Receiving emotional, financial, and instrumental assistance were respectively more important for older people to get better prepared than for younger adults, which is consistent with the socioemotional selectivity theory. Our findings directed attention to the strengths and resilience of older adults during COVID-19 from a life course perspective and highlighted the importance of social support and social relationship in their post-disaster recovery and ongoing preparedness.

